# Evidence of misuse of nonparametric tests in the presence of heteroscedasticity within obesity research

**DOI:** 10.12688/f1000research.52693.1

**Published:** 2021-05-17

**Authors:** Cynthia M Kroeger, Bridget A Hannon, Tanya M Halliday, Keisuke Ejima, Margarita Teran-Garcia, Andrew W Brown

**Affiliations:** 1 Charles Perkins Centre, Central Clinical School, The University of Sydney, Sydney, NSW, Australia; 2 Department of Epidemiology and Biostatistics, Indiana University School of Public Health Bloomington, Bloomington, Indiana, USA; 3 Division of Nutritional Sciences, University of Illinois at Urbana-Champaign, Urbana, IL, USA; 4 Abbott Nutrition, Columbus, OH, USA; 5 Department of Health and Kinesiology, University of Utah, Salt Lake City, UT, USA; 6 University of Illinois Extension, Carle Illinois College of Medicine, University of Illinois at Urbana-Champaign, Urbana, IL, USA; 7 Department of Applied Health Science, Indiana University School of Public Health Bloomington, Bloomington, IN, USA

**Keywords:** Nonparametric tests, heteroscedasticity, research rigor, statistical methods, open science, nutrition, obesity

## Abstract

Background:

Classic nonparametric tests (cNPTs), like Kruskal–Wallis or Mann–Whitney U, are sometimes used to detect differences in central tendency ( *i.e.*, means or medians). However, when the tests’ assumptions are violated, such as in the presence of unequal variance and other forms of heteroscedasticity, they are no longer valid for testing differences in central tendency. Yet, sometimes researchers erroneously use cNPTs to account for heteroscedasticity.

Objective:

To document the appropriateness of cNPT use in obesity literature, characterize studies that use cNPTs, and evaluate the citation and public sharing patterns of these articles.

Methods:

We reviewed obesity studies published in 2017 to determine whether the authors used cNPTs: (1) to correct for heteroscedasticity (invalid); (2) when heteroscedasticity was clearly not present (correct); or (3) when it was unclear whether heteroscedasticity was present (unclear). Open science R packages were used to transparently search literature and extract data on how often papers with errors have been cited in academic literature, read in Mendeley, and disseminated in the media.

Results:

We identified nine studies that used a cNPT in the presence of heteroscedasticity (some because of the mistaken rationale that the test corrected for heteroscedasticity), 25 articles that did not explicitly state whether heteroscedasticity was present when a cNPT was used, and only four articles that appropriately reported that heteroscedasticity was not present when a cNPT was used. Errors were found in observational and interventional studies, in human and rodent studies, and only when studies were unregistered. Studies with errors have been cited 113 times, read in Mendeley 123 times, and disseminated in the media 41 times, by the public, scientists, science communicators, and doctors.

Conclusions:

Examples of inappropriate use of cNPTs exist in the obesity literature, and those articles perpetuate the errors *via* various audiences and dissemination platforms.

## Introduction

Concerns have been raised as to whether scientific research generally ^1– 3^ and nutrition and obesity research specifically ^4– 6^ meet modern standards of research rigor and quality. Various suggestions for improvement in conceptualization, design, or analysis, have been published by the National Institutes of Health, National Academies of Sciences, Engineering, and Medicine, and within nutrition and obesity disciplines. ^7– 10^ One domain that requires attention is using statistical procedures in ways that foster valid inferences. Indeed, it is not uncommon in nutrition and obesity research for inappropriate statistical models and procedures to be chosen and implemented, ^4, 11, 12^ which can erode the quality of the science and trust in the field. ^13^

We previously reported on the consequences of one area of statistical error: erroneously using classic nonparametric tests (cNPTs) to test for differences in central tendency ( *i.e.*, means or medians) when heteroscedasticity is present. ^14^ Heteroscedasticity is a difference in statistical dispersion ( *e.g.*, variance, as used here) among groups. Therein, we defined cNPTs as “tests that do not rely on any assumptions about the data having a particular distribution, other than having a finite mean and a finite variance.” Such methods include Kruskal–Wallis, Wilcoxon signed-rank, and Mann–Whitney U-tests, and exclude methods that do not involve explicit resampling ( *e.g.*, bootstrap). We refer readers to our previous paper to learn more about further intricacies on cNPTs, the misconceptions of using nonparametric tests in the presence of heteroscedasticity, why this error can be of particular concern to nutrition and obesity research, and some guidance on how to avoid some errors. ^14^

The objective of the present study was to determine whether misuse of cNPTs in the presence of heteroscedasticity exists in the obesity literature. Specifically, we sought to identify publications where heteroscedasticity was used as a justification for choosing a cNPT. We also aimed to describe the characteristics of included studies, such as design and model type, open science practices adopted by included studies, and the extent to which the studies have been disseminated since publication, in order to understand the context in which this error occurred and its dissemination impact.

## Methods

### Search methods

A pilot search was conducted in Google Scholar (Google LLC) using combinations and permutations of the terms “nonparametric,” “bootstrapping,” “obesity,” “overweight,” and “adiposity”. Herein, we use the word ‘term’ to mean words and phrases (that is, in contrast to a statistical ‘term’ in a model). This search provided evidence of the use of cNPTs in the presence of heteroscedasticity and helped to inform our search terms for this study. We searched the literature for studies 1) reporting obesity outcomes, 2) mentioning use of nonparametric tests, 3) acknowledging the presence or potential for unequal variances, and 4) published in 2017. Our use of the year 2017 coincided with preparing these materials for our tutorial article ^14^ and allows for the follow up period of three years for the assessment of dissemination impact. The citation and dissemination impacts of those articles are current as of December 9, 2020 (see Screening and Data Extraction). Search terms were expanded based on input from J.J. Pionke, a librarian at the University of Illinois at Urbana-Champaign. The PubMed Central platform was used because it allows for full-text searches. Nutrition and obesity outcome search terms were limited to abstracts, whereas the article body was also searched for heteroscedasticity and nonparametric terms. Because heteroscedasticity and nonparametric terms are key inclusion criteria and are often not included in abstracts, this methodological choice was key to improve the feasibility of this study, considering the large number of nutrition and obesity papers published each year. To make our search reproducible, we used the Open Science R package rEntrez (SCR_021062) ^15^ to conduct our search within PubMed Central. The full search strategy can be found in the study repository. ^16^

### Screening and data extraction

Full texts were obtained and assessed for eligibility and data extraction. Articles were considered eligible if they contained an obesity outcome, a heteroscedasticity term ( *e.g.*, heterogeneous variances, unequal variances, or homogeneity of variance), and a nonparametric term ( *e.g.*, Kruskal–Wallis, rank-sum test, or Wilcox test), and were published in 2017. An obesity outcome was defined as a body composition outcome within a study that aimed to address the problem of overweight or obesity. Heteroscedasticity and nonparametric terms were defined by those used for the literature search, which can be found in the study repository. ^16^ Articles were independently screened, and the data independently extracted by two investigators (CMK and either BAH or TMH). Discrepancies were resolved by consensus among CMK, BAH, and TMH.

The following data were manually extracted from each eligible article: study design (intervention or observational), subject type (human, rodent, or other), type of cNPT used, reason for use of heteroscedasticity terms, and whether findings obtained from the cNPT were statistically significant. The reason the authors referenced a heteroscedasticity term was categorized according to Table 1.

**Table 1.** Categories of use of nonparametric tests in the included studies.

Description of Practice	Abbreviation	Validity
Used a nonparametric test; explicitly stated that heteroscedasticity was not present	Correct	Valid
Used a nonparametric test; explicitly stated that the test was used in the presence of heteroscedasticity or to correct for heteroscedasticity	Error	Invalid
Used at least one nonparametric test; no explicit statement of whether heteroscedasticity was tested for or present	No Link	Unclear
Used a nonparametric test; stated that variance assumptions were tested for, but did not explicitly state whether heteroscedasticity was present	Ambiguous	Unclear

To determine whether improper use of a cNPT was potentially avoided and to gain insight on the prevalence of responsible research practices that help to improve the transparency of data analysis, we also extracted the following information from each included article: whether a study was registered in a study registry ( *e.g.*, clinicaltrials.gov), whether a study was preregistered ( *i.e.*, before the start of data collection), whether statistical analysis plans and outcomes were prespecified, and whether the study data or analysis code were described as “publicly available”, “available upon request,” or not.

Open Science R packages were used to automatically extract data on the relative impact of articles containing errors and ambiguity since their publication in 2017 to December 9, 2020. For instance, rAltmetric ^17^ was used to extract the articles’ overall Altmetric Attention Score (Altmetric LLP), their total number of mentions ( *e.g.*, in news outlets, social media, blogs), and the cohorts giving the articles attention ( *e.g.*, members of the public, doctors, scientists, and science communicators). The Altmetric Attention Score is made up of weighted approximations for volume (total number of original mentions), sources ( *e.g.*, mentions in newspaper articles weigh more than blog posts, which weigh more than tweets), and authors ( *e.g.*, articles shared by doctors to other doctors weigh more than automatic posts from an academic journal account). The package rCrossref ^17^ was used to extract the number of times articles were cited in academic literature. The package rEntrez was used to automatically extract publication date to determine the number of days since publication. The full code for the implemented search strategy, automatic data extraction, included studies, manually obtained data, and associated figure generation can be found at our study repository. ^16^

Given the descriptive nature of this study and having not conducted any formal *a priori* power calculations nor establishing any *a priori* hypotheses, results are presented as counts, frequencies, and other summaries, sometimes stratified by cNPT appropriateness or study characteristics. No formal between-group comparisons or statistical inferences are calculated herein.

## Results

**Figure 1.**
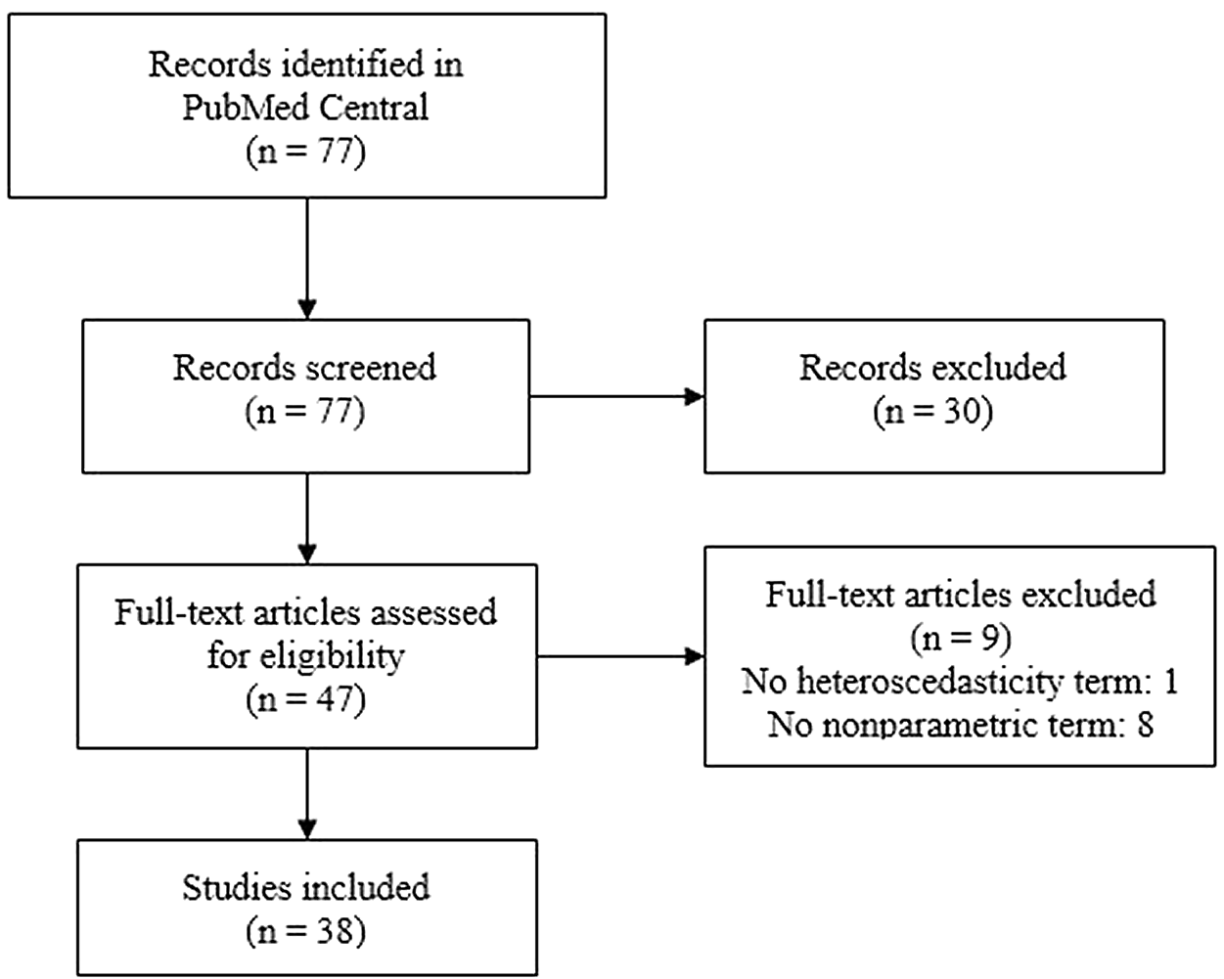
Flowchart of inclusion of studies for review of nonparametric test use.

**Figure 2.**
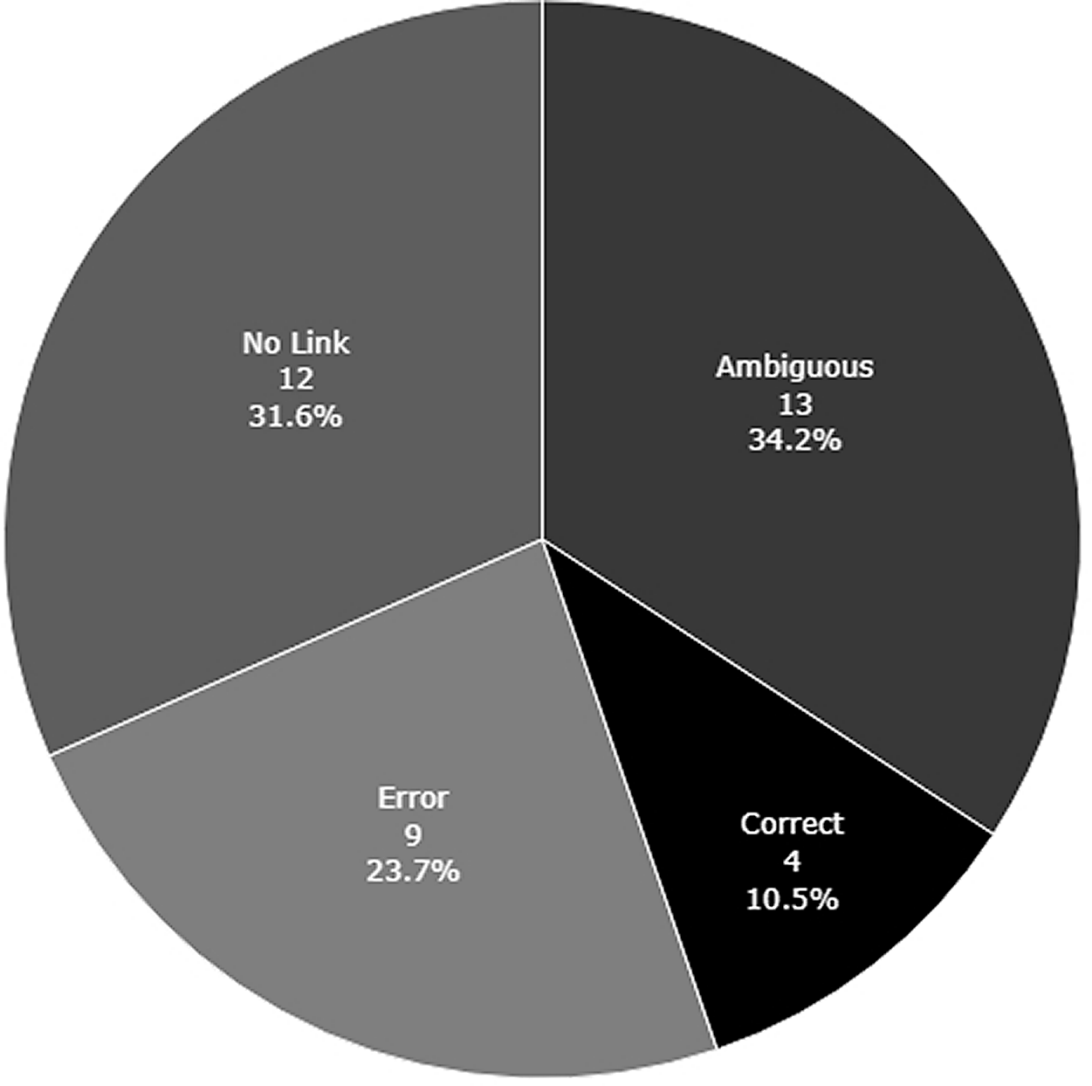
Appropriateness of nonparametric test use in the included studies. Error, used a nonparametric test and explicitly stated that the test was used in the presence of heteroscedasticity or to correct for heteroscedasticity; No Link, used at least one nonparametric test but made no explicit statement of whether heteroscedasticity was tested for or present; Ambiguous, used a nonparametric test and stated that variance assumptions were tested for, but did not explicitly state whether heteroscedasticity was present; Correct, used a nonparametric test and explicitly stated that heteroscedasticity was not present.

Figure 1 depicts the flow diagram of included studies, with 38 ultimately being included. Inclusion of heteroscedasticity and nonparametric terms were not considered if the study did not contain a nutrition obesity outcome or heteroscedasticity term, respectively. Figure 2 shows the proportion of included studies that used cNPTs in clear error, ^9^ ambiguously (25), or correctly. ^4^

**Figure 3.**
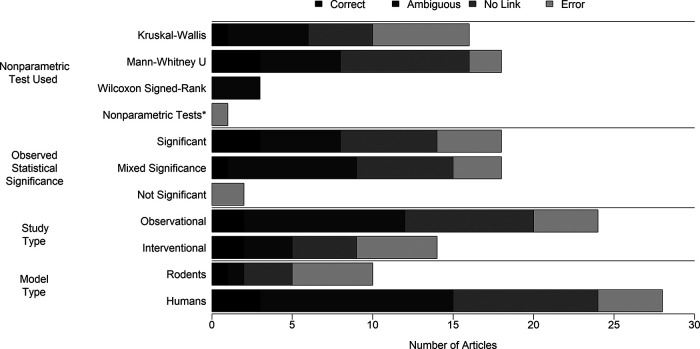
Appropriateness of nonparametric test use in nutrition and obesity studies published in 2017 according to study characteristics. *Nonparametric Tests: Description of the test used was only “non-parametric tests.” Observed statistical significance: Number of articles that report results obtained from a nonparametric test as statistically significant (significant), mixed (mixed significance), or not significant. Correct, used a nonparametric test and explicitly stated that heteroscedasticity was not present; Ambiguous, used a nonparametric test and stated that variance assumptions were tested for but did not explicitly state whether heteroscedasticity was present; No Link, used at least one nonparametric test, but made no explicit statement of whether heteroscedasticity was tested for or present; Error, used a nonparametric test and explicitly stated that the test was used in the presence of heteroscedasticity or to correct for heteroscedasticity.

**Figure 4.**
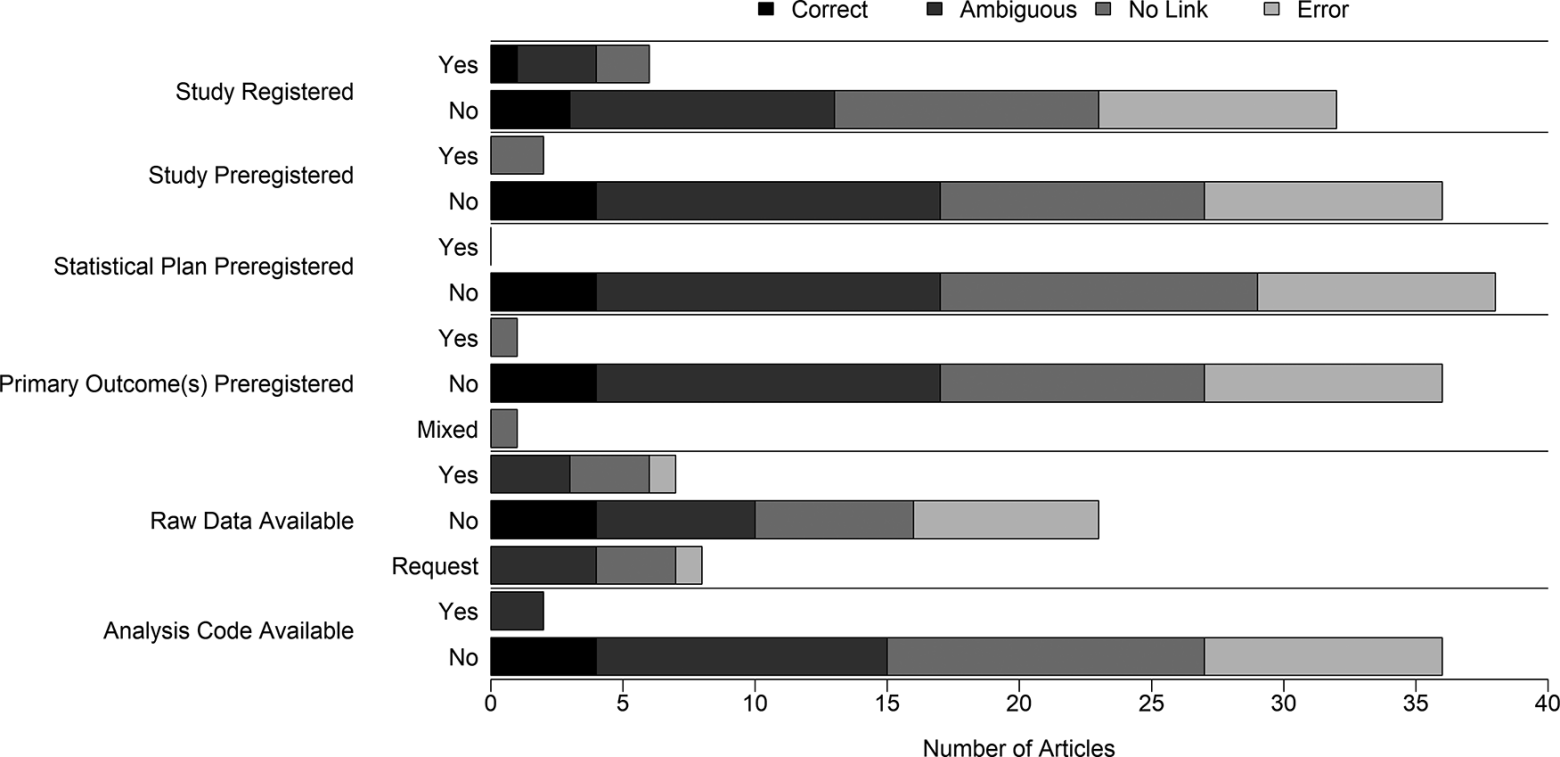
Appropriateness of nonparametric test use in nutrition and obesity studies published in 2017 according to reported responsible research practices. Correct, used a nonparametric test and explicitly stated that heteroscedasticity was not present; Ambiguous, used a nonparametric test and stated that variance assumptions were tested for but did not explicitly state whether heteroscedasticity was present; No Link, used at least one nonparametric test, but made no explicit statement of whether heteroscedasticity was tested for or present; Error, used a nonparametric test and explicitly stated that the test was used in the presence of heteroscedasticity or to correct for heteroscedasticity. For Raw Data Available, “No” means the authors stated the data are not available, the paper had no data availability statement, or the data availability statement was left blank.

A breakdown of study characteristics by appropriateness of cNPT use is depicted in Figure 3. Study designs consisted of interventional (n = 14) or observational (n = 24), and model types of rodents (n = 10) and human participants (n = 28). P-values obtained from the cNPT were statistically significant in n=18 studies, statistically insignificant in n = 2 studies, or a mix of statistical significance depending on the outcome (n = 18). The prevalence of responsible research practices among studies by appropriateness of cNPT use can be viewed in Figure 4. Clear errors were only found in studies that were not registered. Only two studies were preregistered, and no studies preregistered a statistical analysis plan. Raw data were indicated as available for seven studies, including one study with a clear error. Analysis code was only listed as available for two studies but for none of the studies with clear errors.

**Figure 5.**
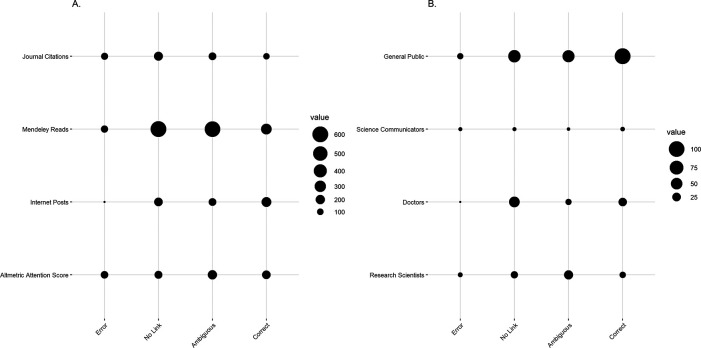
Dissemination of nutrition and obesity studies published in 2017 after an average of 1268 days since publication. A. Total number of journal citations, reads on Mendeley reference library (Mendeley Ltd), internet posts, and total Altmetric Attention Score (Altmetric LLP) by appropriateness of nonparametric test use. Sample size of included articles with dissemination data: Error: n = 4; No Link: n = 11; Ambiguous: n = 9; Correct: n = 4. Citation counts were available for all included studies. **B.** Total number of internet posts (e.g., news, blogs, Twitter, Wikipedia, LinkedIn, Facebook) by cohort and appropriateness of nonparametric test use. Sample size of included articles with dissemination data: Error: n = 4; No Link: n = 11; Ambiguous: n = 8; Correct: n = 4. Correct, used a nonparametric test and explicitly stated that heteroscedasticity was not present; Ambiguous, used a nonparametric test and stated that variance assumptions were tested for but did not explicitly state whether heteroscedasticity was present; No Link, used at least one nonparametric test, but made no explicit statement of whether heteroscedasticity was tested for or present; Error, used a nonparametric test and explicitly stated that the test was used in the presence of heteroscedasticity or to correct for heteroscedasticity.

As of December 9, 2020, the included studies had been cited a total of 531 times across all studies after an average of 1268 days since publication. A total of 323 of these citations were of articles containing ambiguities as to whether heteroscedasticity was present when a cNPT was used, 113 citations were of articles that erroneously used a cNPT to correct for heteroscedasticity or in the known presence of heteroscedasticity, and 95 citations were from articles that transparently reported correct use of nonparametric tests. Altmetrics were available for 28 studies, which had been read and mentioned 1578 and 564 times, respectively, and have received a net Altmetric Attention Score of 634. The cohorts that have been documented engaged in dissemination are the public (n = 225), scientists (n = 62), science communicators (n = 16), and medical doctors (n = 79). Dissemination data are depicted in Figure 5, where they are grouped by appropriateness of cNPT use. Papers with clear errors have been cited in peer-reviewed journals 113 times, read in Mendeley 123 times, obtained an Altmetric Attention Score of 131.8, and shared on the internet by the public (n = 11), science communicators (n = 4), doctors (n = 2), and research scientists (n = 6).

## Discussion

Examples of improper use of cNPTs in nutrition and obesity research were easy to find among articles indexed in PubMed Central in 2017. A common reason for use of cNPTs in statistical analysis was the presence of heteroscedasticity, because assumptions of equal variances were not met. Over half of the included studies also did not clearly state the rationale for use of cNPTs with respect to heteroscedasticity or that heteroscedasticity was not present when a cNPT was used. These studies 1) did not link the rationale for using a cNPT with the use of the heteroscedasticity term, 2) reported that variance assumptions were tested for or met but did not specify for which outcomes or for all cases a cNPT was used, or 3) reported that variance assumptions were tested for or met but did not explicitly state that heteroscedasticity was not present. This lack of transparency in reporting creates ambiguity when interpreting such results, as the reader cannot determine whether a cNPT was used appropriately. The use of inappropriate tests may also lead researchers to draw the wrong conclusion, further muddying the scientific record and increasing mistrust. Without clear explanation of statistical methodology and results, these findings cannot be readily reproduced. Further, because some authors clearly and erroneously used a cNPT with heteroscedasticity, concern is raised as to whether similar misunderstandings are present in cases where reporting is vague, which in our sample occurred in two-thirds of cases. Responsible research practices, such as preregistering statistical analysis plans or clarifying whether data and analysis code are publicly available or available upon request, were not prevalent. Such practices are tools that can help improve replication of statistical findings and make details of the statistical methods more transparent.

Despite containing misused and unclearly used statistical methods, many of the included articles are being cited within both scientific and lay communities. Dissemination of research with improper statistical methods may reinforce the misuse of cNPTs in the presence of heteroscedasticity within the scientific community. If misused or unclearly used cNPTs led to invalid conclusions, both the lay and scientific communities and those they share with can become misinformed of the magnitude or statistical significance of such results.

In some cases, authors explicitly used cNPTs to erroneously attempt to address deviations from homoscedasticity. The use of cNPTs in the presence of unequal variances among experimental groups, especially if sample sizes are imbalanced, will result in an increased type I (false-positive) error rate, as shown using simulations in our previous work. ^14^ These improper statistical methods can lead to incorrect conclusions and dissemination of misleading findings.

### Strengths and limitations

Strengths of our approach include the transparent and reproducible research process. Conducting the literature search programmatically and sharing the code in a study repository allows the possibility of exact replication, as well as clarity around how the search was done. Further, extracting citation counts, date published, and Altmetric data programmatically may help improve the speed of data acquisition while minimizing extraction error that comes with independently manually extracting data. ^18^ Our study repository ^16^ transparently describes the research process for this project. Dissemination data were extracted for a conference presentation in 2018, ^19^ simulations on why this error is a problem statistically were published in 2020, ^20^ and dissemination data were updated for submission of this manuscript and data for peer review publication. The repository explains the differences and reasons for each publication, shows how the dissemination data have changed over time, and foster transparency and reproducibility of each step of the project process. In addition, the use of papers in 2017 is relatively recent to investigate the field’s use of such tests, while also allowing enough lag-time to observe accumulation of dissemination trends.

Limitations of our approach include that we do not know whether our findings are representative of all nutrition and obesity papers, even those published in 2017, but only to those that met our inclusion criteria in PubMed Central. Limiting our review to PubMed Central allowed us to use the R packages “rentrez” and “fulltext” to programmatically search full texts of articles for key inclusion criteria, but excludes studies not indexed in PubMed Central and thus may not be representative. Future work might expand these methods to other databases by using packages to download full texts and text mine them. Furthermore, our approach used terms for heteroscedasticity, nonparametric tests, nutrition, and obesity from a preliminary search of the literature, but may not capture all search terms, and thus may have resulted in some relevant literature being excluded from our review. Consistent with these limitations, we do not present our numbers as evidence of the prevalence of the problem within the literature, but rather as a systematically obtained case series to illustrate issues with cNPT reporting and use within the literature.

## Conclusion

The use of cNPTs in the presence of heteroscedasticity was present among nutrition and obesity articles indexed in PubMed Central and published in 2017. In some cases, the cNPT was erroneously used to correct for heteroscedasticity, while many of the statistical methods and results sections of the included articles were ambiguous. Better reporting and appropriate use of cNPTs is needed in nutrition and obesity literature.

## Data Availability

Zenodo: Underlying data for ‘Evidence of misuse of nonparametric tests in the presence of heteroscedasticity within obesity research’. https://doi.org/10.5281/zenodo.4733330. ^16^ This project contains the following underlying data:
Raw data Code book Analytical code Data are available under the terms of the Creative Commons Attribution-ShareAlike 4.0 International license (CC BY-SA 4.0).
