# Supplementary material for: Evidence of misuse of nonparametric tests in the presence of heteroscedasticity within obesity research
Source: F1000Res. 2021 May 17;10:391. [Version 1] doi: 10.12688/f1000research.52693.1 (PMC8792877; doi:10.12688/f1000research.52693.1)
Supplement: Peer review history

# Peer review: Reviewer response for version 1

**DOI:** 10.5256/f1000research.56003.r120824

**Published:** 2022-01-26

**Authors:** Rand R Wilcox

**Affiliations:** 1 Department of Psychology, University of Southern California, Los Angeles, California, USA

Yes, these classic methods are indeed inappropriate for dealing with measures of central tendency under general conditions.  This has been known in the statistics. literature for many years. This paper underscores the fact that these insights remain unknown by many researchers. Generally, there is a serious gap between advances relevant to basic techniques and how the typical researcher is trained. Hopefully this paper will help motivate others to deal with this problem.

 There is a vast literature dealing with robust methods for making inferences about measures of central tendency (Wilcox (2022 ^1^)). They deal with heteroscedasticity as well as concerns about non-normality. It might help readers if this is pointed out.

Is the work clearly and accurately presented and does it cite the current literature?

Yes

If applicable, is the statistical analysis and its interpretation appropriate?

Yes

Are all the source data underlying the results available to ensure full reproducibility?

No source data required

Is the study design appropriate and is the work technically sound?

Yes

Are the conclusions drawn adequately supported by the results?

Yes

Are sufficient details of methods and analysis provided to allow replication by others?

Yes

Reviewer Expertise:

Robust statistical methods

I confirm that I have read this submission and believe that I have an appropriate level of expertise to confirm that it is of an acceptable scientific standard.

## References

[1] : Introduction to Robust Estimation and Hypothesis Testing. *Academic Press* .2022;

# Peer review: Reviewer response for version 1

**DOI:** 10.5256/f1000research.56003.r99209

**Published:** 2021-11-10

**Authors:** Joscha Krause

**Affiliations:** 1 Department of Economic and Social Statistics, Trier University, Trier, Germany

**Short summary:**

 The presented paper provides a literature review on the use of nonparametric tests for the detection of central tendency differences in grouped obesity research data. In particular, the authors investigate how often corresponding tests have been applied in the presence of heteroscedasticity. Since many common tests (e.g., Kruskal-Wallis, Mann-Whitney-U) assume variance homogeneity between groups, their application in such circumstances can lead to false conclusions. To evaluate how common such misuse is in the literature, the authors review articles published in 2017. They evaluate whether the nonparametric tests were applied correctly, incorrectly, or whether it cannot be determined.

**Comments:**

- Being a statistician, I can only comment on the statistical aspects of the presented paper.
- The paper is compact and well-written. It is well-known that a significant number of empirical studies in this field suffer from inaccuracies due to a lack in statistical rigor. Accordingly, a quantitative evaluation providing evidence on this problem is a welcome contribution.
- The authors give a transparent description on how they searched for relevant papers, and how they categorized the studies. They further provided the necessary programming code for reproduction. The presentation of the results is clear and expedient. The authors point out that their study is purely descriptive and does not provide any statistical inference. Against this background, there is not much room for methodological points of critique. Within this scope, the study is well done. 
- Maybe the authors would like to consider the subsequent minor points as a proposal for some additions to the manuscript.
   
  - For readers from statistics, it would have been interesting to see how exactly a violation of the homogeneity assumption affects the test outcomes. An increase in type-1-errors is mentioned. However, is this due to the asymptotic distributions of the test statistics being invalid, are the tests biased, do they lose testing power? I understand that this paper is a ‘follow-up’ to a previous technical study on this matter by the authors. Nevertheless, maybe they can add a few more words on that aspect. 
  - Another interesting point would have been to see how often the false application of the tests in the literature has actually led to seriously wrong conclusions. However, even without that aspect, the presented paper makes a convincing case for addressing this important issue. 

**Recommendation:**

- I have no serious concerns from a statistical perspective and, thus, approve the indexing of the paper.

Is the work clearly and accurately presented and does it cite the current literature?

Yes

If applicable, is the statistical analysis and its interpretation appropriate?

Yes

Are all the source data underlying the results available to ensure full reproducibility?

Yes

Is the study design appropriate and is the work technically sound?

Yes

Are the conclusions drawn adequately supported by the results?

Yes

Are sufficient details of methods and analysis provided to allow replication by others?

Yes

Reviewer Expertise:

Statistics, data science, Monte Carlo simulations

I confirm that I have read this submission and believe that I have an appropriate level of expertise to confirm that it is of an acceptable scientific standard.
